# Exploration of the Changes in Facial Microbiota of Maskne Patients and Healthy Controls Before and After Wearing Masks Using 16 S rRNA Analysis

**DOI:** 10.1007/s44197-024-00240-6

**Published:** 2024-05-21

**Authors:** Kexin Deng, Xiaofei Tong, Shuyue Chen, Guojun Wu, Ke Shi, Hao Chen, Yurong Tan, Junlin Liao, Jianda Zhou, Junxiang Zhao

**Affiliations:** 1 Department of Burn and Plastic Surgery, The Third Xiangya Hospital, Central South University, No. 138, Tongzipo Road, Yuelu District, Changsha City, Hunan Province China; 2 Department of Burn and Plastic Surgery, Nanshi Hospital Affiliated to Henan University, No. 1081, Zhongzhou West Road, Nanyang City, Henan Province China; 3 School of Basic Medical Science, Central South University, No. 172, Tongzipo Road, Changsha City, Hunan Province China; 4 Center of Burn & Plastic and Wound Healing Surgery, The First Affiliated Hospital, Hengyang Medical School Hengyang, NO.69, Chuanshan Road, Hengyang, Hunan China

**Keywords:** Microbiota, Maskne, Acne, Skin, Mask, 16s rRNA

## Abstract

Whether in the field of medical care, or in people’s daily life and health protection, the importance of masks has been paid more and more attention. Acne, the most common complication after wearing masks, which is also called maskne, has been successfully introduced into the common language as a common topic of dermatologist consultations. This study aims to study the changes of microflora in maskne patients and healthy controls before and after wearing masks. In the summer of 2023, we collected a total of 50 samples from 15 maskne patients and 10 healthy controls before and after wearing surgical masks for a long time. 16 S ribosomal DNA sequencing and identification technology with V3-V4 variable region were adopted to explore the microbiome changes caused by mask wearing, analyze the changes in microbial diversity, and make interaction network. LDA effect size analysis was used to identify which bacteria showed significant changes in their relative abundance from phylum to genus. After wearing a mask, the microbiome of the maskne patients changed significantly more than that of the healthy controls, with both α diversity and β diversity lower than those of maskne patients before wearing masks and those of healthy controls after wearing masks. Co-occurrence network analysis showed that compared with other groups, the network of maskne patients after wearing masks for a long time had the lowest connectivity and complexity, but the highest clustering property, while the opposite was true for healthy controls. Many microbes that are potentially beneficial to the skin decreased significantly after wearing a mask. There was almost no difference in healthy controls before and after wearing a mask.

**Supplementary Information:**

The online version contains supplementary material available at 10.1007/s44197-024-00240-6.

## Introduction

Acne is a chronic inflammatory disorder of the pilosebaceous unit. It is the eighth most common disease worldwide, with a prevalence of 9.4% [1]. Globally, about 85% of individuals aged 12–24, 8% of adults aged 25–34, and 3% of adults aged 35–44 suffer from acne [2, 3]. In addition to severe physical burdens such as painful abscesses and unsightly scars, it can also lead to psychological problems such as depression [4], create economic burdens, and increase unemployment rates [4]. The clinical characteristics of acne include excessive sebum secretion, non-inflammatory lesions (open or closed comedones), inflammatory lesions (papules and pustules), and varying degrees of scarring. Acne lesions are most common in areas with a high density of pilosebaceous units, such as the face, neck, upper chest, shoulders, and back. In some cases, nodules and cysts can lead to severe nodulocystic acne [5]. The main factors that lead to acne are as follows: Excessive sebum secretion leads to the accumulation of excess oil on the skin. Abnormal follicular keratinization results in the disordered shedding of epithelial cells, causing blockage of the sebaceous ducts. Excessive inflammatory response and secretion of inflammatory factors. Altered distribution of bacteria. Bacteria associated with acne mainly include *Propionibacterium acnes*, *Staphylococcus epidermidis*, *Malassezia spp*., and *Staphylococcus aureus* [6]. Shi et al.’s study showed that acne patients exhibited lower α-diversity in their facial microbiome, indicating that the skin microbiome of acne patients had fewer types and was less uniform than that of healthy controls [7].

*Lachnospiraceae*, *Clostridiales*, *Moraxellaceae*, *Prevotella*, and *Lactococcus garvieae* were the top 5 most abundant species found in patients with acne using 16sRNA sequencing, but were not found in healthy controls [7]. *Achromobacter*, *Stenotrophomonas*, *Porphyromonas*, *Prevotella*, and *Pseudomonas* were the top 5 most abundant species in healthy controls, but were not found in patients with acne [7]. Furthermore, there was a correlation between the integrity indicators of the epidermal barrier and the skin microbiota in acne patients [8]. In comparison to healthy controls, acne patients often exhibited increased trans-epidermal water loss (TEWL) and reduced diversity in the microbiota [8]. Shannon and Simpson diversity index were used for quantitative analysis of skin microbiota diversity, and it was found to be negatively correlated with the severity of acne and TEWL [8]. These findings highlight the close involvement of the microbial community in the pathological changes observed in acne patients.

Since the early 21st century, with several global outbreaks of respiratory infectious diseases, the meaning of masks has permeated every aspect of daily life. Whether in healthcare settings or personal health protection, the importance of mask usage has been increasingly emphasized. However, the prolonged use of masks has brought facial skin issues. According to previous studies, the most common skin adverse reactions associated with mask wearing were acne, followed by itching symptoms and rashes [9, 10].

Maskne is a newly coined term derived from mask-related acne, which is a type of mechanical acne resulting from the continuous textile–skin adherence and friction [11]. Maskne has become a common topic of discussion among dermatologists and has been successfully integrated into everyday language. There are over 2,000,000 hashtags related to maskne on social media platforms [11]. The diagnostic criteria for maskne include:

I. Acne occurs within 6 weeks after using the mask or the original acne in the mask area worsens [12, 13];
II. Elementary lesions such as papules, pustules, and comedones;
III. Mask area positioning;
IV. Temporal relationship with mask use: aggravation/development of acne with prolonged usage (> 4–6 h/day) and improvement when not worn for a long period [14];
V. Exclude other dermatoses, such as perioral dermatitis, rosacea, seborrheic dermatitis, irritant contact dermatitis, allergic contact dermatitis [12];

Maskne is essentially acne issues related to mask wearing. It has been observed that acne patients exhibit noticeable facial microbiota imbalance and reduced microbial diversity [7]. The underlying pathology of maskne is thought to by caused by the microclimate formed beneath the skin covered by the mask. This microclimate results in elevated temperature, increased humidity, reduced air circulation, toxin accumulation and excessive sebum secretion, leading to changes in the skin microbiota and acne development [15–17].

However, at present, there is a lack of specific research on skin microbiota changes among acne patients or maskne sufferers before and after wearing a mask. The specific changes in skin microbiota diversity and the compositional differences in microflora from the phylum to genus levels between maskne patients and healthy controls before and after mask usage remain unclear. Therefore, this study aims to utilize 16 S rRNA sequencing technology to uncover the alterations in skin microbiota of maskne patients and healthy controls before and after wearing a mask. By exploring the changes in facial microflora, the study seeks to elucidate the potential reasons behind acne caused by prolonged mask wearing in maskne patients. This study helps address concerns of health care workers and others who need to wear masks for long periods of time about mask complications, such as maskne, and facilitates research on facial microbiome balance.

## Method and Materials

### Volunteers Recruitment and Preparation Before Sample Collection

Maskne patients and healthy controls aged 16–25 years living in Hunan Province, China were recruited from June 1, 2023 to October 1, 2023, and facial microflora samples were also collected during this period. The average temperature during the study period is about 23–26 °C, the average maximum temperature is about 33–38 °C, and the relative humidity is about 70–85%. Participants who had lived in Hunan for several years, had not not been treated with antibiotics or use other medications that may affect facial flora in the three months prior to sampling, and were willing to avoid using any other medications during the test was recruited. After a three-month washout period, participants were asked to choose a test time point that did not overlap with their menstruation for sample collection, and they were given a week to prepare before sample collection. Written informed consent was obtained from each participant before registration. All procedures involving human participants in this study adhered to the principles of the Helsinki Declaration. The study has been approved by the Institutional Review Board of the Third Xiangya Hospital, Central South University (Protocol Number: [21,141]).

#### Inclusion Criteria

1. Residing in Hunan Province, China.
2. Patients:

Patients were classified as moderate or severe according to the Global Acne Grading System (GAGS) during the period when they were experiencing maskne, regardless of their current skin condition. Before collecting facial microbiome swab samples, individuals diagnosed with maskne completed a three-month washout period. The purpose of setting the washout period is to eliminate and reduce some events or behaviors in daily life that may have an impact on the skin flora and reduce bias. Under the supervision of medical staff, during this period, they are not allowed to wear masks of any material, including surgical protective masks, KN95, cotton masks, etc., and they are not allowed to use any drugs that affect the skin flora, such as salicylic acid, Adapalene gel, asidifuran cream, glucocorticoids, various antibiotics, etc. Only those who met the above conditions were finally collected.

Healthy controls:

Individuals without acne or any other skin diseases, such as atopic dermatitis, allergic dermatitis, eczema, etc. They also went through a washout period, and only those who met the above conditions were finally collected.

3. Age between 16 and 25 years.

#### Exclusion Criteria

Participating in other clinical studies in the past three months; Pregnant or lactating women; presence of any skin disease (e.g., atopic dermatitis, psoriasis, bruises, eczema, urticaria, rosacea, etc.); Deformities or tattoos in the study area or open wounds, use of medications within the past three months, including antibiotics, hormonal medications, retinoids, adapalene, alpha hydroxy acids, beta hydroxy acids, salicylic acid, tretinoin, or other skincare products with known efficacy in treating acne or skin were excluded.

#### Preparation Before Sample Collection

After the washout period, the volunteers were given a week for preparation before the official sample collection and should avoid swimming in chlorinated pools, as well as using hot water, saunas, or ultraviolet for sterilization. Taking into account the time required for the microbiome to return to balance after a routine facial cleansing regimen, the patient did not undergo any facial cleansing procedures such as facial cleanser, makeup remover oil, or even water in the 24 h prior to the collection of the facial swab. During the 12 h before the official start of facial swab collection, participants should take special care not to allow any objects to come into contact with the face, such as tissues, glasses, fingers, etc.

### Samples Collection

We collected skin swab samples from the covered area of the surgical mask before wearing the mask at 2pm and from the unduplicated covered area after wearing the mask for 4 h at 6pm.Using a sterile skin swab moistened with physiological saline, random samples were collected from the cheek area within the surgical mask-covered region. The swab sample covered an area of 2.5 × 2.5 cm². During the collection process, the swab head was alternately wiped on the skin 25 times in a cross mode, each collection took about 30 s, and the wiping force was controlled to make the facial skin sag about 1 cm. Swabs were placed in 1.5 ml phosphate-buffered saline and immediately stored at -20 °C before DNA extraction. Once all the samples are collected, we move on to DNA extraction, PCR amplification, and sequencing.

In order to express conveniently, we abbreviate healthy controls before and after wearing masks to NB and NA, and abbreviate maskne patients before and after wearing a mask to B and A in the following paper.

### DNA Extraction, PCR Amplification, and Sequencing

Skin swabs were taken out and placed in 2 ml EP tubes with 500 µl of lysis buffer, 10 µl of beta-mercaptoethanol and 20–50 µl of Proteinase K. The samples were then vortexed in a 56 °C water bath for 2–16 h, and the swabs were discarded. Next, 700 µl of DNA extraction solution (phenol: chloroform: isoamyl alcohol = 25:24:1) was added and thoroughly mixed by inversion. The mixture was centrifuged at 9000 rpm, 4 °C for 15 min. The supernatant was collected, and an equal volume of isopropanol was added, gently mixed by inversion, left to stand on ice or in a -20 °C freezer for 10 min, and then centrifuged at 12,000 rpm, 4 °C for 10 min. The supernatant was discarded, and the white precipitate was washed with 1 ml of cold 70% ethanol, gently mixed by inversion, and centrifuged at 12,000 rpm, 4 °C for 5 min. This washing step was repeated, and then the supernatant was discarded, leaving the white precipitate to air dry residual ethanol. An appropriate volume of ddH2O was added, and the concentration was measured using a micro UV spectrophotometer. The extracted DNA was subjected to 1.2% agarose gel electrophoresis to detect the genomic DNA.

The 16S V3-V4 region sequences were selected for high-throughput sequencing analysis. The library was constructed by two-step PCR amplification. Using purified DNA as a template, PCR amplification was performed using 16S V3-V4 region universal primers 357F 5’-ACTCCTACGGRAGGCAGCAG-3’ and 806R 5’-GGACTACHVGGGTWTCTAAT-3’, which included barcodes and fusion primers for sequencing. The PCR products were analyzed by 1.2% agarose gel electrophoresis, and the samples with good detection results were excised from 2% agarose gel for recovery. All PCR products were recovered using the AxyPrep DNA Gel Recovery Kit (AXYGEN USA), quantified with the FTC-3000TM Real-Time PCR System (Fengling, Shanghai), mixed in equimolar ratios, and subjected to an additional 8 cycles of PCR amplification. Illumina platform sequencing adapters, sequencing primers, and barcodes were added to both ends of the target fragments to complete library construction. The constructed libraries were sequenced using the Novaseq 6000 SP 500 Cycle Reagent Kit (Illumina USA) at Microbiota Technology (Shanghai) Co., Ltd.

For the first PCR reaction: 5x Buffer 10µL, dNTPs (10 mM) 1µL, Phusion High-Fidelity DNA Polymerase 1U, forward and reverse primers (10 µM) each 1µL, template DNA 20–50 ng, and ultra-pure water to a total volume of 50µL. The PCR conditions were: 94 °C for 2 min; then 94 °C for 30 s, 56 °C for 30 s, 72 °C for 30 s, and finally 72 °C for 5 min, a total of 24 cycles.

For the second PCR reaction: 5x Buffer 8µL, dNTPs (10 mM) 1µL, Phusion High-Fidelity DNA Polymerase 0.8U, forward and reverse primers (10 µM) each 1µL, template DNA 5µL, and ultra-pure water to a total volume of 40µL. The PCR conditions were the same as the first reaction, with a total of 8 cycles.

### Data Processing

Sequencing was conducted by Tinygene Biotech Co., Ltd. (Shanghai, China). The obtained paired-end reads (PEreads) were initially sorted based on the barcodes of each sample. Subsequently, the sequence quality control and filtering were performed. The sequences were then merged according to their overlap relationships, then the quality control and filtering were carried out again. The specific steps are outlined below:

Sequence quality control using Trimmomatic (version: 0.38): If the average quality score within the window was less than 20, the bases starting from the window were pruned away from the end, and reads below 50 bp after filtering were filtered. The sliding window approach was employed, and the window size was 50 base pairs (bp).

Adapter and primer trimming using cutadapt (version: 1.16): cutadapt software was used to process sequencing adapters and primers.

Sequence Merging using FLASH (version: 1.2.11): PE reads were merged into a single sequence using FLASH, taking into account the overlap relationship between PEreads. The minimum overlap length was set to 10 bp, and the maximum allowable mismatch rate in the overlap region was 0.2. Additional parameters were set as follows: maxambig = 0, maxhomop = 8, minlength = 200, maxlength = 485. Merged sequences that didn’t meet the criteria were filtered out.

The resulting optimized sequences were subjected to Operational Taxonomic Units (OTUs) clustering analysis and taxonomic classification. USEARCH (version: 8.1.1861) software was used to cluster the assembled sequences into OTU. The main steps were as follows:

Utilizing UPARSE, a representative sequence for each OTU was obtained by clustering at the 97% sequence similarity level. Sequence comparison between UCHIME and the existing chimeric database golddatabase (v20110519) was performed to remove the chimeric sequences generated by PCR amplification from the representative sequences of OTUs. The usearch_global method was employed to align all sequences back to the representative sequences of OTUs, thus generating abundance tables for every OTU in each sample. Following the acquisition of representative OTU sequences, mothur (classify.seqs, version: 1.39.5) software was used to annotate these sequences with species information by aligning them against a reference database. The confidence threshold of 0.6 was set for the annotations. The databases used for alignment were Silva128 (bacteria) and Silva119 (archaea). OTUs without annotation results or annotated to species not relevant to the analysis project were filtered out. For example, if the analysis project focuses on bacterial 16 S samples, the OTUs annotated as archaea is removed. The software used for this step includes USEARCH (version: 8.1.1861) and mothur (version: 1.39.5).

### Statistical Analysis

All statistical analyses were performed in R V3.6.0 environment. Without special instructions, the statistical results were visualized using the “ggplot2” package. Alpha diversity was measured using the function “diversity” in the package “Vegan” based on the flat taxonomy table. Gini-Simpson diversity index was obtained by subtracting the value of the classical Simpson index from 1. Beta diversity was compared using principal coordinate analysis (PCoA) based on Bray-Curtis distances. Redundancy analysis (RDA) was also conducted using Vegan. Beta diversity across sample groups was compared by PERMANOVA with permutations of 999. ANOSIM test was used to evaluate the inter-group significance. According to the criteria of Wang et al., *R* > 0 and *p* < 0.05 were considered to be statistically significant. The relative abundance data at the genus level (excluding unclassified) were divided into groups to construct a separate network map. The “cor.test” function from R version 3.6.3 was employed to compute the Spearman correlation coefficients, and the p-values was corrected by Benjamini-Hochberg (BH) method. Species pairs with correlation coefficients above 0.8 and p-values below 0.05 were selected to establish correlations. Network graph visualization used the “ggraph version 2.0.3” package, with the “group_components” function to determine the modules within the network. Nodes and edges within the same Module were assigned the same color. The “centrality_degree” calculated degrees, the “triangles” calculated local_triangles, and the “group_edge_betweenness” computed Cluster attributes. Visualization involved the use of “ggplot2 version 3.4.2” and “ggsignif version 0.6.0” to create box plots, with the wilcox.test method assessing inter-group differences. The “centrality_hub” computed hub_score attributes and selected the Module with the highest hub_score value as the hub_network for visualization.

In addition, biomarkers of the sample groups were discovered by Linear Discriminant Analysis (LDA) Effect Size (LEfSe)3. The strategy for multi-class analysis was set one-against-all, and the threshold on the logarithmic LDA score for discriminative features was set to 3.0.

It should be emphasized that A and B, NA and NB are paired sample groups.

## Results

### Background Characteristics of the Study Cohort

This study included 15 patients with maskne and 10 healthy controls, whose background characteristics were shown in Table 1. The participants ranged in age from 18 to 25 years old; the average age was 19.4 and 19.8 years old in the maskne and healthy controls, respectively. In the patients group, there were 9 cases of moderate acne, 8 cases of severe acne during their maskne period. A total of 50 skin swabs were collected, which were distributed as follows: 15 samples obtained from maskne patients before wearing masks, 15 samples obtained from maskne patients after wearing masks, 10 samples obtained from healthy controls before wearing masks, 10 samples obtained from healthy controls after wearing masks. All skin samples met the standard for analysis. No participants withdrew from the study, and no data was lost.

**Table 1:** Demographics of patients with acne and healthy controls

Factors	Normal	Acne	*P* value
Sex (F/M)	10(5/5)	15(7/8)	
Age, years, mean ± SD	19.8 ± 2.4	19.4 ± 2.1	0.97

### 16sRNA Sequencing Quality

A total of 3,287,779 clean reads were obtained, with an average of 53,190 reads per sample after removing unqualified sequences. Shannon index analysis showed that the sequencing depth covered rare new phylotypes and most microbial diversity (Online Resource Fig.**S1**a). The species accumulation curve exhibited a trend of sharp increase at first and then gradual increase, indicating that the sampling was sufficient. With the increase of sample size, the species addition rate decreased. This trend suggests that the sample size of the system is sufficiently representative for the species composition of the system (Online Resource Fig.S1b).

### Changes in Facial Microbiome Diversity Before and After Wearing Masks

**Fig. 1:**
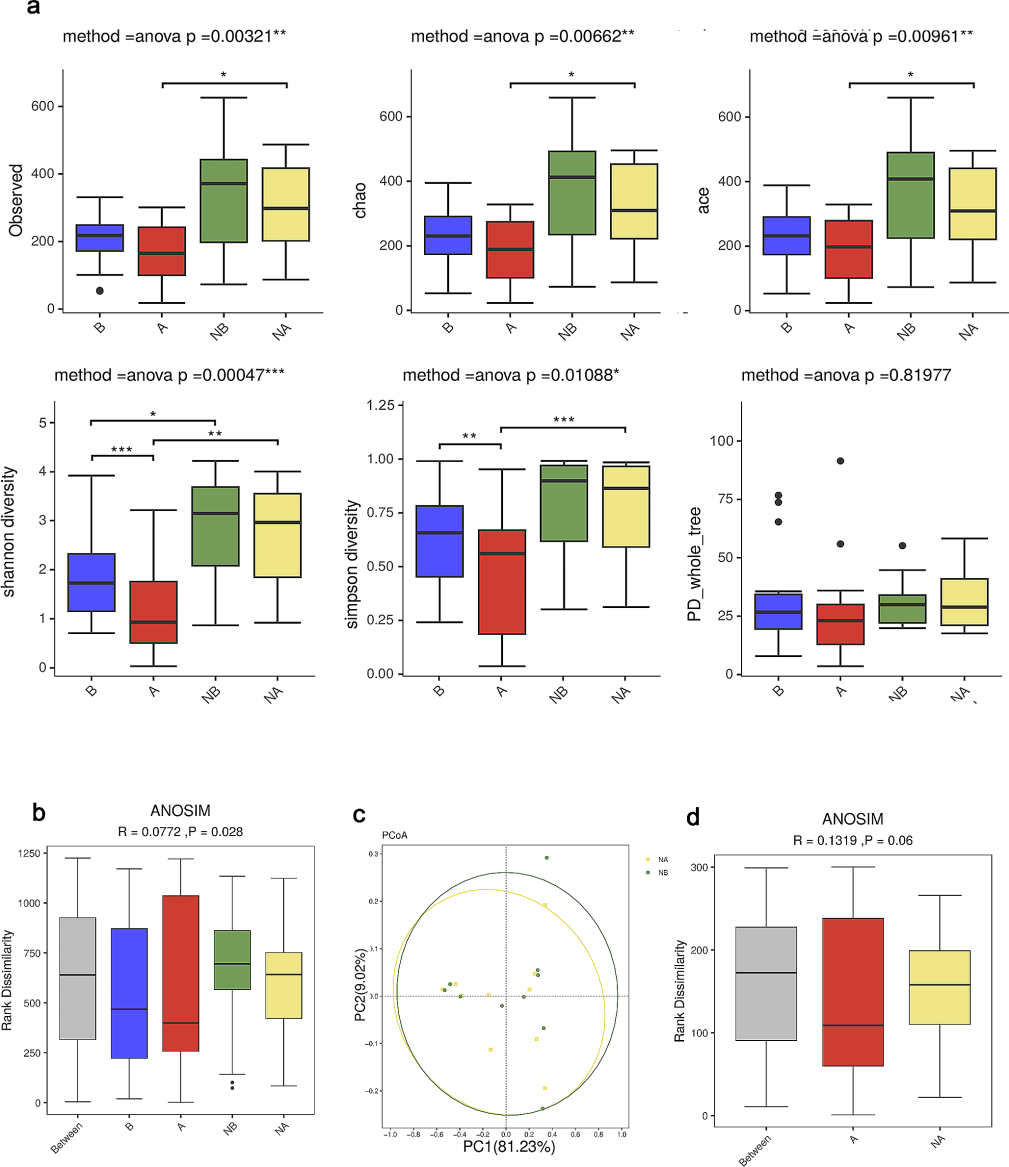
Changes of microbial diversity (**a**) α diversities include observed diversity, Chao diversity, Ace diversity, Shannon diversity, and Gini-Simpson diversity. Paired sample groups: A and B, NA and NB were analyzed with the paired ANOVA t-Test, Non-paired sample groups: A and NA, B and NB were analyzed with ANOVA t-Test. *p < 0.05;**p < 0.01; ***p < 0.001. (**b**) ANOISM demonstrated significant differences among A-B-NA-NB groups using Bray-Curtis distance. (**c**) Principal coordinate analysis (PCoA) of NA and NB groups using Bray-Curtis distance. (**d**)ANOISM demonstrated statistical differences among A-NA groups using Bray-Curtis distance (*p* < 0.1)

Alterations in facial microbiome diversities before and after wearing masks were examined among maskne patients. As depicted in Fig. 1a and Online Resource File.S1, it was evident that the α-diversities of the microbiome after wearing masks among maskne patients were notably lower compared to those of healthy controls. It includes observed diversity (*P* = 0.0096, α = 0.05), Chao diversity (*P* = 0.0192, α = 0.05), Ace diversity (*P* = 0.0357, α = 0.05), Shannon diversity (*P* = 0.0036, α = 0.005), and Gini-Simpson diversity (*P* = 0.0096, α = 0.005). There were also substantial alterations in α-diversity among maskne patients before and after wearing masks, particularly with a significant reduction in Shannon diversity (*P* = 0.0036, α = 0.005) and Gini-Simpson diversity (*P* = 0.0096, α = 0.005) after wearing masks. In contrast, there was no significant difference in microbial diversity before and after wearing masks among healthy controls (α = 0.05). However, when comparing healthy controls with maskne patients before wearing masks, only Shannon diversity exhibited significant difference (*P* = 0.041, α = 0.05), which was consistent with previous literature reports [7] By comparison of principal coordinate analysis (PCoA) and Bray-Curtis distance ANOSIM test (*R* = 0.07, *p* < 0.05), there were significant differences in Beta diversity among all groups (Fig. 1b). From Fig. 1c and d, it can be seen that the NA and NB groups almost coincide, but the ANOISM test shows a slight difference between the A group and the NA group (*P* < 0.1).

**Fig. 2:**
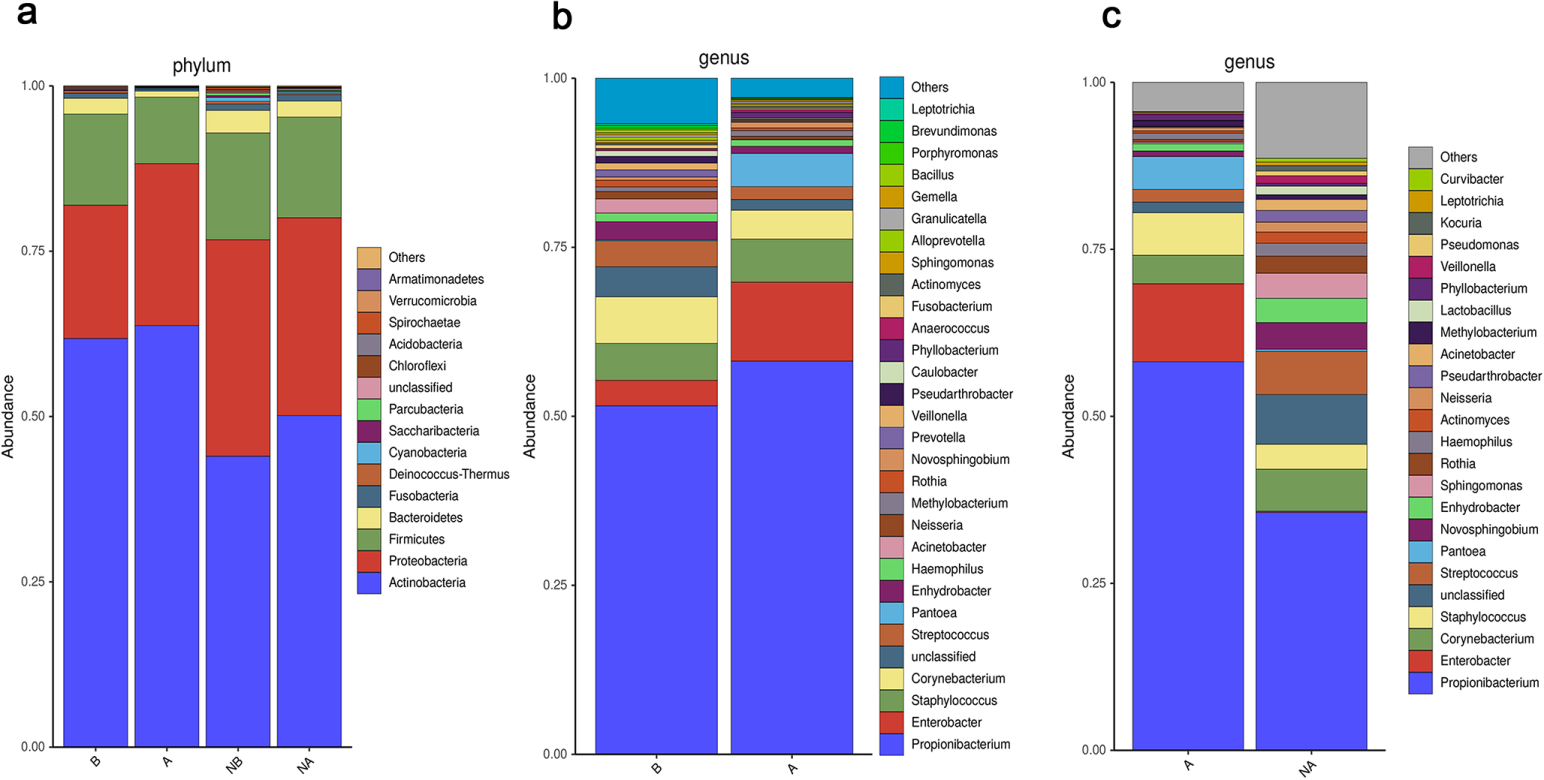
Composition of bacterial communities at the phylum and genus levels (**a**) Composition of the microbiomes of the four groups at the phylum level; (**b**) Comparison of bacterial community composition at the genus level between groups A and B (**c**) Comparison of bacterial community composition at the genus level between groups A and NA. **A**: Maskne patients after wearing masks for a long time. **B**: Maskne patients before wearing masks for a long time. NA: Healthy controls after wearing masks for a long time. NB: Healthy controls before wearing masks for a long time

As depicted in Fig. 2a, at the phylum level, among the top 10 abundant bacterial phyla in maskne patients, the microbial communities showed significant reductions in Bacteroidetes (*p* = 0.0125, α = 0.05) and Fusobacteria (*p* = 0.026, α = 0.05) after wearing a mask, while Chloroflexi exhibited a significant reduction after wearing a mask (*P* = 0.002, α = 0.05) among the top 10 abundant phyla in healthy controls. However, no significant difference was observed at the phylum level between maskne patients and healthy controls after wearing masks. As illustrated in Fig. 2b, at the genus level, among the top 30 abundant bacterial genera in maskne patients, there is a statistically significant decrease after wearing a mask in the genera *Corynebacterium* (*P* = 0.021, α = 0.05), *Enhydrobacter* (*P* = 0.005, α = 0.05), *Acinetobacter* (*P* = 0.033, α = 0.05), *Rothia* (*P* = 0.035, α = 0.05), *Veillonella* (*P* = 0.012, α = 0.05), *Brevundimonas* (*P* = 0.011, α = 0.05), *Leptotrichia* (*P* = 0.028, α = 0.05), and *Paracoccus* (*P* = 0.038, α = 0.05). No statistically significant change was observed in the top 30 abundant genera among healthy controls after wearing a mask. However, when comparing maskne patients and healthy controls after wearing masks, significant decreases were observed in the genera *Actinomyces* (*P* = 0.036, α = 0.05), *Pseudarthrobacter* (*P* = 0.039, α = 0.05), *Acinetobacter* (*P* = 0.020, α = 0.05), and *Pseudomonas* (*P* = 0.046, α = 0.05) (Fig. 2c).The related data was shown in Online Resource Tab.S1.

### Analysis of Microbial Networks in Maskne Patients and Healthy Controls Before and After Wearing Masks

**Fig. 3:**
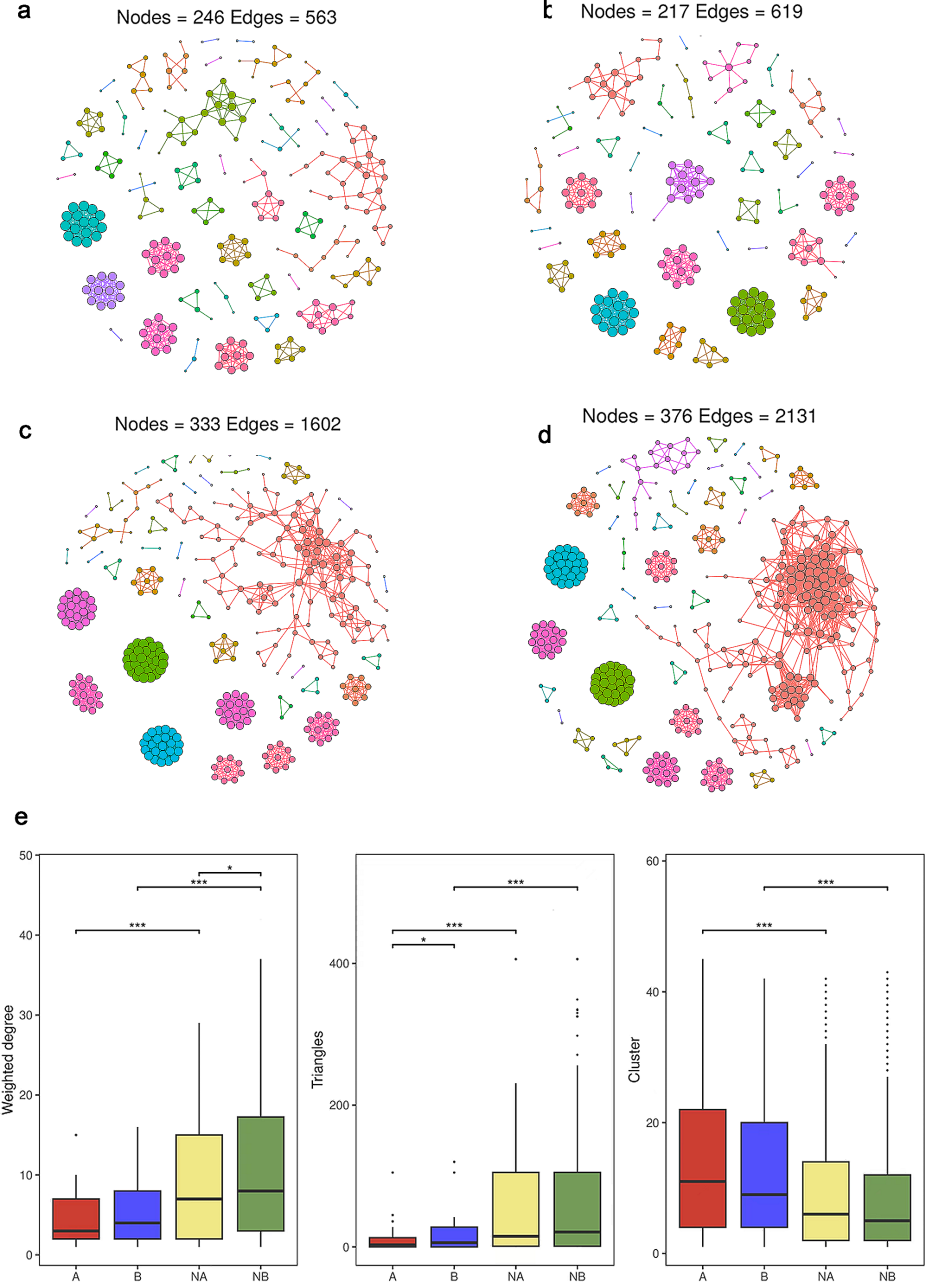
Co-occurrence network in four groups (**a**) Network of maskne patients after wearing mask (A), (**b**) Network of maskne patients before wearing mask (B), (**c**) Network of healthy controls after wearing mask (NA) and (**d**) Network of healthy controls before wearing mask (NB); Each different color in Fig. 3a, b and c, and 3d represents a module within the network. Nodes, which represent different genera, are grouped into these modules, with nodes within the same module having more connections among themselves and fewer connections with nodes in other modules.(**e**) Comparison of network topology properties among groups, weighted degree, triangles, and cluster. Wilcoxon test, *p < 0.05;**p < 0.01; ***p < 0.001

There are complex interactions among different microbial species in microbial community, such as symbiosis, competition and coexistence. By analyzing the co-occurrence relationships among microorganisms (whether they co-occur or not) in microbiome data, co-occurrence network analysis can reveal the interaction patterns within microbial communities and help to depict the complex structure of microbial communities [18]. We performed a network co-occurrence analysis to unravel the relationships among microorganisms. Under the same network construction parameters, the network of maskne group after wearing masks (Fig. 3a) had 246 nodes and 563 edges, the group of maskne (Fig. 3b) had 217 nodes and 619 edges before wearing. There were 333 nodes and 1602 edges in normal group network after wearing masks (Fig. 3c), with 373 nodes and 2131 edges before(Fig. 3d). In addition, A group and B group were clustered into 45 and 42 modules respectively, while NA and NB groups were 39 and 41 (Online Resource Tab.S2). The results showed that microbial networks were composed of tightly connected nodes and formed a kind of “small-world” topology (Online Resource Tab.S2; Online Resource Fig.S2). Accordingly, we also analyzed the network properties of each group of networks. The average degree of internal connectivity reflected in group A and NA was 4.577 and 9.622, respectively, and that in group B and NB were 5.705 and 11.335, respectively, both higher than that in their paired groups (Fig. 3e), but only the difference between NA and NB groups was statistically significant (*P* < 0.05). The difference between A and NA groups was also significant (*P* < 0.001). The triangles formed in B and NB groups were also higher than those of their paired groups (Fig. 3e), but only the difference between A and B groups had statistic significance (*P* < 0.05), and the triangles formed between A and NA groups were also significant (*P* < 0.001). These suggested that total connectivity and complexities about facial skin microbiomes in groups B and NA were higher than in group A. The NB group was also higher than its paired NA group. The network clusters of groups A and NA were higher than those of paired groups B and NB respectively (Fig. 3e), but there was no statistic significance yet, while the cluster attribute of group A was significantly higher than that of NA(*P* < 0.0001). These results manifested that the average “clustering property” of the whole networks within facial skin microbiomes in groups A and NA were higher than in paired groups B and NB respectively, as well as the difference between groups A and NA. Co-occurrence network analysis in microbial community research is a method to explore microbial interactions.

To gain insight into each of the four groups, we extracted the microbial hub network. Among the four groups of hub networks, group A had the highest abundance of *criinalium* (Online Resource Fig.S1S3a), Group B had the highest abundance of *Hansschlegella* (Online Resource Fig.S3b), and group NA had the highest abundance of *Methylotenera* (Online Resource Fig.S3c). The abundance of *Crossiella* in NB group was the highest. (Online Resource Fig.S3d). In general, NB group had the largest number of nodes and the highest degree of agglomeration. These results indicate that there are differences in facial microbial interaction networks in groups A, B, NA and NB. Compared with other groups, Group A network has the lowest connectivity and complexity, and the highest clustering.

### LEfSe Analysis of the Four Groups

**Fig. 4:**
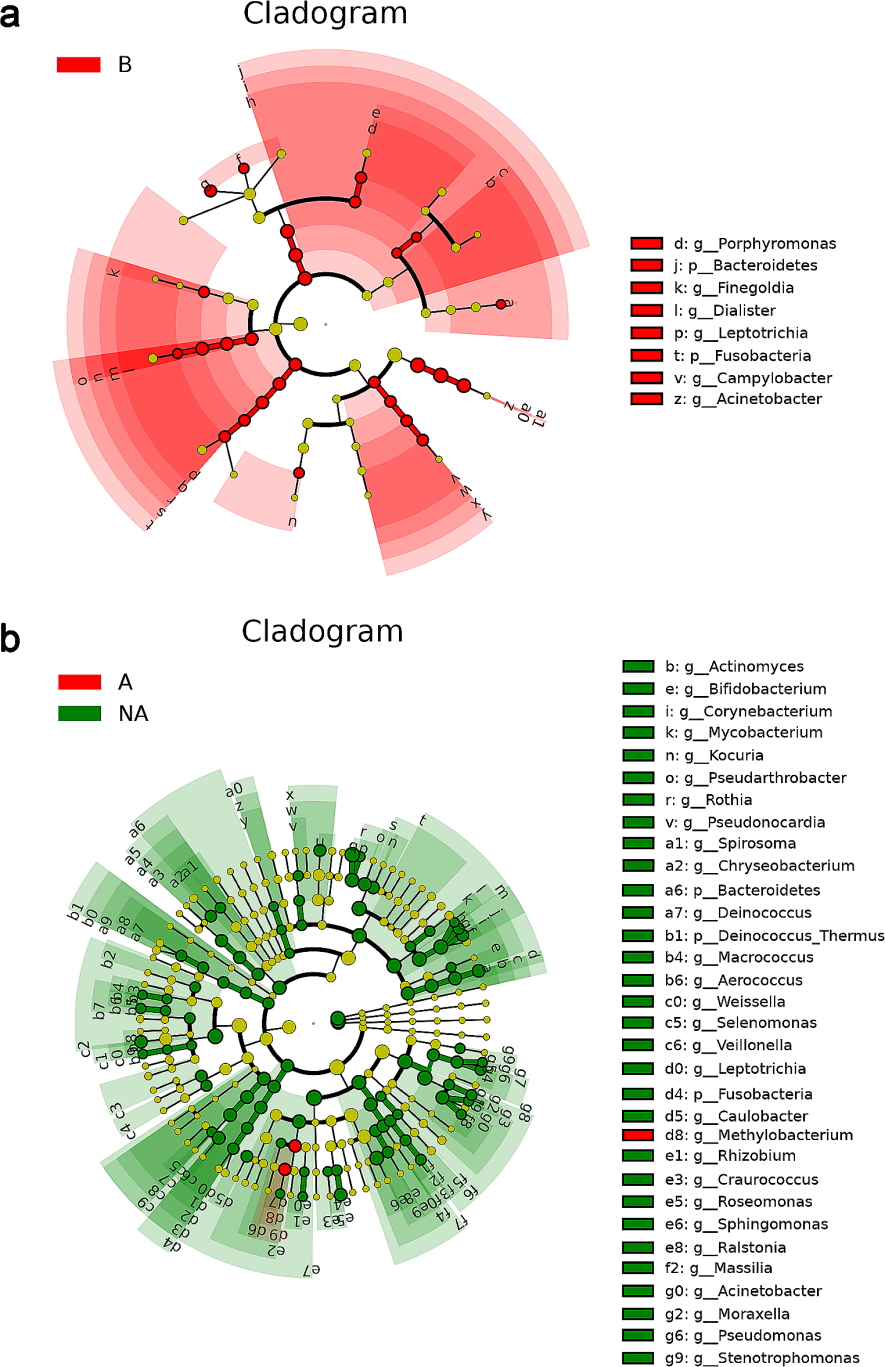
LEfSe analysis of taxonomy with significant differences in abundance among groups Evolutionary branching diagram: the circles radiating from the inside to the outside represent taxonomic levels from the phylum to the genus. Each small circle at different taxonomic levels represents a taxon at that level, and the diameter size of the small circles is proportional to the relative abundance size. (**a**) Cladogram between A and B. Species without significant differences are uniformly hided or colored in chartreuse, with red nodes representing microbial taxa that play an important role in group B. The names of the species indicated by letters in the figure are shown in the legend on the right. (**b**) Cladogram between A and NA. Species without significant differences are uniformly hided or colored in chartreuse, with red nodes representing microbial taxa that play an important role in group A, and green nodes representing microbial taxa that play an important role in group NA. The names of the species indicated by letters in the figure are shown in the legend on the right. A: Maskne patients after wearing masks for a long time. B: Maskne patients before wearing masks for a long time. NA: Healthy controls after wearing masks for a long time. NB: Healthy controls before wearing masks for a long time

From the above results, it can be seen that the change of facial microbiomes of the maskne group before and after wearing mask is significantly greater than that in healthy controls, and there are also significant differences in the microbiota between group A and group NA. Thus, LEfSe analysis was performed to explain which bacteria had significant changes in their relative abundances from phylum to genus. The threshold of adjusted P and LDA score were 0.05 and 3. The findings demonstrated that the *phyla Bacteroidetes* and *Fusobacteria* were the most significantly altered ones, and at the genus level, *Acinetobacter*, *Campylobacter*, *Leptotrichia*, *Porphyromonas*, *Finegoldia*, *Dialister* had significant decreases in their relative abundances in group A compared to their paired B group (Online Resource Fig.S4a). As for A and NA groups, *Bacteroidetes*, *Fusobacteria*, *Deinococcus_Thermus* were significant in group NA with increased relative abundances at phylum level. (Online Resource Fig.S4b). Meanwhile, *Sphingomonas*, *Rothia*, *Corynebacterium*, *Acinetobacter*, *Pseudarthrobacter*, *Actinomyces*, *Veillonella*, *Kocuria*, *Pseudomonas*, *Chryseobacterium*, *Caulobacter* genus were significantly associated with group NA, and *Methylobacterium* was significantly associated with group A at the genera level. For groups NA and NB, there was no change at the phylum and genera level when the LDA score was 3. The relative abundances of the genera *Acinetobacter* related to groups B and NA dropped almost to 0% in group A (Online Resource Tab.S3). In terms of relative abundance, *Acinetobacter*, *Rothia*, *Pseudarthrobacter*, *Veillonella*, *Actinomyces* were the most 5 altered genera in groups A and NA. The cladogram showed the most relevant clades among groups, which was in accordance with the above results (Fig. 4a and b).

## Discussion

### Determination of some Experimental Conditions and Parameters in this Study

For patients already suffering from acne, it has been reported that wearing masks for extended periods (> 4 h) exacerbates acne symptoms, and intensifies facial discomfort, such as itching, stinging, redness, and swelling [19]. Prolonged mask wearing time (> 4 h), mask type, mask reuse frequency and wearing frequency have been demonstrated to be significantly associated with acne exacerbation [19, 20]. In addition, surgical masks are the most widely used and common mask type globally [9]. Therefore, in this study, volunteers wore medical surgical masks for 4 h to collect facial swabs.

### Comparison with Previous Researches

Based on the findings of this study, the α-diversity and β-diversity of maskne patients significantly decreased after wearing masks compared to their pre-mask-wearing state and the healthy controls after mask-wearing. This is contrary to the previous study by Wongtada et al., which showed that wearing a mask had no significant effect on the skin microbiota in patients with mild acne vulgaris [21]. We hypothesized that this notable discrepancy from prior research was due to differences in acne severity selected for the present analysis. Specifically, we focused on patients with moderate to severe acne, while previous studies examined individuals with mild acne. Additionally, they used different facial regions for their microbial controls, while we performed a before-and-after comparison in the area covered by the mask. Prior to the beginning of our experiment, we implemented a strict mask-free period to establish baseline conditions. Furthermore, the effect of mask-wearing on the facial microbiota of healthy controls was negligible, aligning with the findings of Hillebrand et al., who concluded that mask-wearing did not induce changes in the facial microbiota of healthy controls [22] Moreover, in our study, the bacteria primarily discovered using 16 S rRNA technology were *Cutibacterium acnes*, *Corynebacterium*, and *Staphylococcus*, similar to previous research where the bacteria present in masks after wearing were primarily *Cutibacterium acnes* and *Staphylococcus*.

### Possible Roles of some Microorganisms that Varied at the Phylum and Genus Levels

T-test and LefSe were used to look for changes in bacteria in maskne patients and healthy controls after wearing masks for a long time.

We observed that in healthy controls, only the *phylum Chloroflexi* decreased after wearing masks. Although there hasn’t been sufficient exploration regarding Chloroflexi [23], it is worth noting that in probiotic-based skin therapies, its increased abundance is associated with improved skin conditions [24]. Moreover, the *phylum Chloroflexi* and the genera *Brevundimonas* have been identified as significant microbial components in some spring waters known that promote skin regeneration [25].

After wearing a mask, maskne patients exhibited significant decreases in the *Corynebacterium*, *Enhydrobacter*, *Acinetobacter*, *Rothia*, *Veillonella*, *Brevundimonas*, *Leptotrichia*, *Paracoccus*, *Campylobacter*, *Porphyromonas*, and *Finegoldia* at the genus level. A positive correlation of Enhydrobacter with various skin physicochemical parameters such as stratum corneum hydration has been shown [8]. *Veillonella*, *Leptotrichia*, and *Campylobacter* have not been studied enough in relation to the skin. Some species of *Paracoccus* exhibited increased abundance after facial cleansing [26] and produced potent antioxidant astaxanthin [27]. In facial microbiota of acne and pyoderma patients, *Porphyromonas* is nearly absent. Finegoldia is enriched in healthy controls compared to rosacea patients. Interleukin-2 receptor is negatively correlated with *Dialister* as a predictor of psoriasis activity [28].

Compared to healthy controls after wearing masks, maskne patients exhibited significant reductions in the abundances of *Bacteroidetes* and *Fusobacteria* at the phylum level after mask-wearing. *Bacteroidetes* has been found to significantly decrease in adverse skin conditions [29], and in atopic dermatitis, the abundances of *Bacteroidetes* and *Fusobacteria* decreased significantly with increasing disease severity [30]. At the genus level, there were notable decreases in *Streptococcus, Actinomyces, Pseudarthrobacter, Acinetobacter, Pseudomonas, Sphingomonas, Kocuria, Chryseobacterium, Caulobacter, Veillonella*, and *Rothia*. Certain species of *Streptococcus* produce bacteriocin-like inhibitory substances (BLIS) that inhibit *Propionibacterium acnes* growth [31] and secrete ceramides promoting improvement in atopic dermatitis [32], preventing skin aging [33]. There seems to be an antagonistic relationship between *Actinomyces* and *Propionibacterium acnes*, as *Actinomyces viscosus* supernatant demonstrating lytic activity against *Propionibacterium acnes* growth cells [34]. Some strains of *Pseudomonas* possess immunomodulatory and anti-biofilm capabilities [35, 36], with a potential role in skin repair [37, 38]. An extract from *Sphingomonas* can alleviate the negative effects of senescence in human skin [39]. *Kocuria* has a protective effect on the skin [40] and is significantly reduced in inflammatory lesions [41]. *Chryseobacterium* can produce a unique bacterial pigment called *Flexirubins*, which is used in the treatment of chronic skin diseases like eczema [41]. Research in the field of *Pseudarthrobacter, Caulobacter, Veillonella, and Pseudomonas* in dermatologic field remains limited.

In maskne patients after wearing masks, the quantities of *Acinetobacter* and *Rothia* were significantly lower than pre-mask-wearing patients and healthy controls. Certain species of *Acinetobacter* in the skin microbiota have been found to protect against allergic sensitization and inflammation [42]. Additionally, research has shown that certain types of *Acinetobacter* on mucosal surfaces increased after surgery and were associated with improved quality of life in chronic rhinosinusitis [43]. Topical ozone therapy has been found to restore microbiome diversity in atopic dermatitis, leading to an increased abundance of *Acinetobacter* [44]. *Rothia* has been proved to prevent skin aging and possess certain anti-inflammatory properties, which may be related to its short-chain fatty acids [45, 46].

After wearing a mask, *methylobacterium* was significantly increased in maskne patients compared to healthy controls. *Methylobacterium* is a highly lipophilic opportunistic pathogen that primarily exists in soil and plants. It possesses strong adhesive and biofilm-forming characteristics, and has the ability to withstand high temperatures (50–60 °C) [47]. However, no studies have linked *Methylobacterium* to skin diseases. Some studies have found a significant association between *Methylobacterium* and antibiotic-induced dysbiosis [48]. Therefore, *Methylobacterium*, as a newly emerging genus, warrants further investigation into the interaction between itself and acne or skin conditions.

It is worth mentioning that we found no significant changes in *Propionibacterium acnes* in this study. The proliferation of *Propionibacterium acnes* has been closely associated with the occurrence and development of acne. However, recent studies have shown that the abundance of *propionibacterium acnes* does not differ between acne patients and healthy people [49], nor does its abundance correlate with the severity of the disease [49]. The severity of acne is characterized by specific strains of *C*. *acnes* [50]. A study identified the top 10 genotypes (RT1-RT10) of *Propionibacterium acnes* with the highest abundance in acne patients and healthy controls, and found that the top 3 genotypes, RT1, RT2, and RT3, were evenly distributed in both acne and normal follicular sebaceous units [51]. However, the RT4 and RT5 were significantly enriched in up to 40% of acne individuals, but were rarely found in healthy controls. In contrast, RT6 was enriched in 99% of healthy skin individuals [51]. RT4 and RT5 were classified as IA-2 type clades, possesses certain virulence and were closely associated with acne inflammation [50]. However, current 16 S rRNA technology cannot accurately classify specific phylotypes of *Propionibacterium acnes*.

### Discussion on Network Analysis

Nowadays, a wealth of evidence suggests that changes in the microbiome are related to acnes. Dysbiosis of the facial microbiome is reflected not only in the change of the abundance of the microbiome components, but also in the change of the microbial interaction relationship. Many studies have pointed out that there are widespread competitions between bacteria in addition to cooperation in networks [52]. In the analysis of Co-occurrence network, we found that after wearing masks, the internal connectivity of the microbiota significantly decreased in maskne patients compared to healthy controls. Additionally, the internal connectivity of the skin microbiota after wearing masks in maskne patients was also noticeably lower than that in healthy controls. These results indicate that wearing masks has a significant negative impact on the structural stability and complexity of the facial microbiota, leading to a reduction in the interactions between internal bacteria. The weighted degree and the triangle showed similar trends in network analysis, while the cluster exhibit opposite trend. The weighted degree represents the sum of the connection strengths between nodes, and the triangle represents the closed triangle structures formed by three nodes in the network [53]. In microbiome studies, the number of triangles can reflect the complex interactions between microbial species, that is, more triangles usually indicate more complex and dense coexistence pattern [18]. Clusters indicate the strength of larger community formation in the network [18] Therefore, we can observe that wearing masks reduces the connectivity strength within the facial bacteria, decreases complex interaction relationships, increases the degree of clustering, and simplifies the structure of the microbiota, which is detrimental to the normal microsystem’s ability to resist biological or physicochemical invasions from the external environment [54]. Based on the results of the network analysis, we speculate that wearing a mask reduces the self-regulatory stabilization function within the microbiome and negatively affects its normal interaction with human skin, which requires further specific research in the future.

### Limitations

However, current 16 S rRNA technology cannot accurately classify specific phylotypes and species, which is a limitation of our study. In the future, we will study the relationship between changes in the abundance of different Propionibacterium acnes phylotypes and mask-wearing. We will also employ more precise experimental techniques, such as metagenomics technology, to study at the species level.

In addition, there are other shortcomings in this study. Because of the small sample size, although many of the bacteria closely associated with acne changed in number, these changes were not statistically significant enough to draw meaningful conclusions. Moreover, the experimental period was mainly in the summer, which could have been influenced by factors like temperature and humidity. Furthermore, the study was conducted in a limited population from central China, so future studies are expected to explore larger, more diverse cohort to provide broader insights.

## Conclusion

In conclusion, our research provided evidence for the imbalance of facial microbiome of maskne patients, clarified the differences in the facial microbiome among groups NA, NB, A and B, elucidated the changes of facial microbiome on maskne patients before and after wearing masks. Among them, the microbiota in group A was significantly dysfunctional and the diversity of microbiota was the lowest. Network analysis showed different network connectivity, complexity and aggregation degree, among which the network connectivity of group A was significantly sparser and the complexity was the lowest. T-test and LefSe were used to search for bacteria that have changed in maskne patients and healthy controls after wearing masks for a long time. The changes in phylotypes of *Propionibacterium acnes* are still worth exploring in maskne patients after wearing masks. This study has identified specific microbial changes in acne, the most common complication caused by prolonged mask wearing. This finding can assist researchers and manufacturers in enhancing mask manufacturing processes.

## Electronic Supplementary Material

Below is the link to the electronic supplementary material.

Supplementary Material 1

Supplementary Material 2

## Data Availability

Data is stored in https://www.ncbi.nlm.nih.gov/bioproject/PRJNA1017432, and the project is PRJNA1017432.

## Abbreviations

GAGS: Global Acne Grading System
PEreads: Paired-end reads
OTUs: Operational Taxonomic Units
PCoA: Principal coordinate analysis
RDA: Redundancy analysis
LDA: Linear Discriminant Analysis
LEfSe: Effect Size
TEWL: Trans-epidermal water loss
BLIS: Bacteriocin-like inhibitory substances
RTs: Genotypes
